# Ethnopharmacology for Skin Diseases and Cosmetics during the COVID-19 Pandemic in Lithuania

**DOI:** 10.3390/ijerph19074054

**Published:** 2022-03-29

**Authors:** Zivile Pranskuniene, Rugile Grisiute, Andrius Pranskunas, Jurga Bernatoniene

**Affiliations:** 1 Department of Drug Technology and Social Pharmacy, Lithuanian University of Health Sciences, LT-50162 Kaunas, Lithuania; jurga.bernatoniene@lsmuni.lt; 2 Institute of Pharmaceutical Technologies, Lithuanian University of Health Sciences, LT-50162 Kaunas, Lithuania; rugile.grisiute@stud.lsmu.lt; 3 Department of Intensive Care Medicine, Lithuanian University of Health Sciences, LT-50161 Kaunas, Lithuania; andrius.pranskunas@lsmuni.lt

**Keywords:** ethnopharmacology, skin diseases, cosmetics, COVID-19, Lithuania

## Abstract

The documentation of ethnopharmaceutical knowledge has always been important for the preservation of countries’ cultural, social, and economic identity. The COVID-19 pandemic with the collapse of healthcare, which has left the individual health to self-care, has also forced us to look back at ethnopharmacology from a practical point of view. This is the first study in Lithuania, dedicated entirely to ethnopharmaceuticals used for skin diseases and cosmetics, and the first study to analyse ethnopharmacology as a Lithuanian phenomenon during the ongoing COVID-19 pandemic. The main purpose of this study was to collect and evaluate ethnopharmaceutical knowledge regarding skin diseases and cosmetics in Šiauliai District, Lithuania during the COVID-19 pandemic from July 2020 to October 2021. This study surveyed 50 respondents; the survey was conducted using the deep interview method. The respondents mentioned 67 species of medicinal plants from 37 different families used for skin diseases (64.18%), cosmetics (13.44%) and cosmeceuticals (22.38%). Of the 67 plant species, 43 (64%) were not included in the European Medicines Agency monographs and only 14 species (21%) of all included species were used with European Medicines Agency approved medical indications for skin diseases. In terms of public health, the safety of “self-treatment” and recovery rituals for skin diseases are no less important than ethnopharmacological knowledge and its application, this being especially relevant during the COVID-19 pandemic.

## 1. Introduction

Lithuanian scientists first viewed ethnopharmacology as a cultural phenomenon. It was analyzed in historical, ethnocultural and geographical contexts. Traditional folk medicine is an area of folk culture which includes medicinal knowledge, beliefs, and treatments that existed in traditional rural communities [1]. It is necessary to document traditional knowledge since many communities are losing their cultural, social, and economic characteristics [2].

Lithuanian medics, interested in ethnopharmacology, are viewing it not only as a part of the Lithuanian history of medicine, but also as a significant opportunity for medical science and practice. The use of ethnopharmaceuticals, analyzed from both ethnological and ethnomedical aspects, has forced us to view ethnopharmacology as a whole entity. An opinion that ethnopharmacology is not only a way of treatment but also the means of health care and disease prevention has formed [3,4]. 

Currently, the interest in ethnopharmacological research is not only noticeable in developing countries but in the developed world as well. The COVID-19 pandemic with the collapse of healthcare, which has left the health of individuals to self-care, has forced us to look back at ethnopharmacology from a practical point of view [5].

The World Health Organization has estimated that more than 6 billion people depend on plant-based and animal-based medicine. Various populations have natural, widely used pharmacopoeia, where animal and plant derived ingredients are used in the preparation of modern medicines, herbal, and traditional medicines [6]. In the 21st century, medicinal plants have not lost their significance in human lives. Their application value for prophylactic and treatment purposes keeps increasing. The inhabitants of various countries actively make use of local and imported plants and their parts. As many as 80% of the world’s medicinal products are made from medicinal plants, consequently the research of plants is still relevant. Today, as in the past, there are still specialists in phytotherapy since the knowledge and analysis of plants to this day remains a relevant issue.

One of the areas of research and application of ethnopharmacology is dedicated to very important human organ, the skin. The European Medicines Agency, as well as the World Health Organization and the European Scientific Cooperative on Phytotherapy, have confirmed that one of the most frequent indications for which many medicinal plants are used in the European Community and in the rest of the world, is the treatment of skin disorders and minor wounds [7]. Researchers around the world are researching plants and looking for natural means to treat skin diseases and create cosmetics [8,9,10,11]. 

Skin care and preventative treatments have a strong impact on the condition of skin, consequently scientists pay a lot of attention to cosmetics and cosmeceuticals (the active and science-based cosmetics). Natural agents are gaining popularity nowadays as most women prefer natural products for their personal care. These products supply the body with nutrients and enhance health and at the same time are free from synthetic chemicals and have relatively less side-effects compared to synthetic cosmetics. Following this tendency, more medicinal plants are used for the development of new drugs, cosmeceuticals, and pharmaceutical applications [12]. The investment in ethnobotanical studies can minimize the loss of knowledge and, with specialized analytics and more complex in vitro skin models, can develop and help better understand the effects that plants have on human skin [7]. In addition, preventative cosmetic procedures can reduce psychological disorders that may increase in patients during the COVID-19 pandemic situation [13]. 

Studies have shown that during the COVID-19 pandemic, patients were concerned with their appearance and continued to undergo cosmetic procedures and pay attention to their skincare [14]. The emotional health and well-being of a person heavily depends on aesthetic appearance, therefore the disrupted availability of cosmetology services due to the pandemic was a stressful occurrence in Lithuania as well. This is the first study in Lithuania dedicated entirely to skin diseases and cosmetic ethnopharmaceuticals, and the first study to analyze ethnopharmacology as a phenomenon in Lithuania during the ongoing COVID-19 pandemic. The main purpose of this study was to collect and evaluate ethnopharmaceutical knowledge regarding skin diseases and cosmetics in the Šiauliai District during the COVID-19 pandemic in Lithuania.

## 2. Materials and Methods

### 2.1. Study Area

The climate of Lithuania is transitional between the continental type in the East and the oceanic type of Western Europe. Therefore, here predominate air masses influenced by the Atlantic alternating with continental Eurasian or Arctic air masses. The coldest month in Lithuania is January with an average temperature of −5 °C; the warmest month is July with an average temperature of 17 °C. The average annual rainfall is about 800 mm [15]. Climate change also affects Lithuanian vegetation, which inhabits several different regions. The maritime region is dominated by pine forests, the sand dunes by shrubby plants, spruce dominate in the hilly eastern part, oaks in the central part, birches, black alder, aspen in the north, while pine forests are prevalent in the south. About 1/3 of the vegetation consists of forests, 1/5 is meadows, and a small part is wetlands. Dzūkija is the most forested ethnographic area in Lithuania, while the least forested is Suvalkija. Coniferous trees make up 56% of Lithuania’s forests, while deciduous trees make up 39% and hardwoods 4%. The average age of forests in Lithuania is from 50 to 69 years [16]. 

The regions in Lithuania that have formed throughout the course of history are called ethnographic regions. Their boundaries roughly coincide with the boundaries of Lithuanian dialects. The regions differ in their internal structure, cultural traditions, architecture, dialects, and activities [17]. Samogitia is a unique ethnographic region of Lithuania, first mentioned in the Volhynian Chronicle in 1219. It stands out from other homesteads in terms of planning, landscaping, work tools, household utensils, landscape, traditions, and dialect. The traditional dishes of Samogitia are cibulynė, or blood sausage with groats, and boiled potatoes with hemp. Samogitians have a reputation for being very stubborn, sincere, unhurried, and hospitable people. Samogitia is characterized by these craftsmen: carpenters, shoemakers, tailors, weavers, wheelers, and blacksmiths. Pottery is especially popular. Samogitia used to be famous for its wooden chapels, chapel columns, crosses, and figurines of saints [18]. 

**Figure 1:**
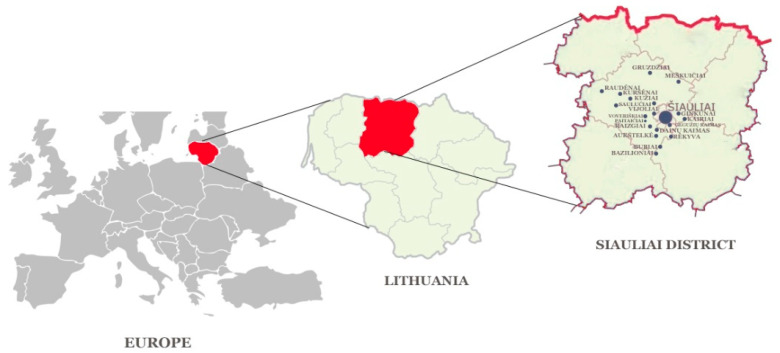
Study area.

Šiauliai district is in the Northwest of Lithuania, located directly on the border between Aukštaitija and Samogitia, although it is considered a part of Samogitia. The district covers 1807 square kilometres, consists of 11 elderships, with a population density of 26 people/sq. km. The district has 1 city, 7 towns and 521 villages within its territory. The highest point is Grinikai hill (183.4 m). Šiauliai district is in the middle plain of the Venta river, in the lowland of the Mūša Nemunėlis and in the Eastern Samogitian plateau. Forests cover 34.8% of the district’s territory. Spruce and birch forests are predominant. The most important extractable minerals in the district are gravel and sand [17,18]. Most of the respondents lived closer to the border of Samogitia (Kuršėnai, Daugėliai, Kužiai, Gruzdžiai, Lukšiai, etc.) (Figure 1). Although the Samogitian dialect is quite prevalent in Samogitia, people who can speak the dialect usually use it only with people they are close to with, in a private environment and less often in public. Although the respondents were mostly from the Samogitian side of the border, they did not use the dialect during the interviews. 

### 2.2. Methods

The study was conducted from July 2020 to October 2021 in Šiauliai district, in the territory of the Samogitia ethnographic region (Figure 1). The purpose of this study was explained to each interviewee and an informed consent form was signed prior to the study. The study was conducted in accordance with the Code of Ethics of the International Society of Ethnobiology [19]. The research was approved by the Bioethics Centre of the Lithuanian University of Health Sciences (No. BEC-FF-30). The study surveyed 50 respondents, 9 men (18%) and 41 women (82%). The respondents were mostly farmers, housewives and even medical professionals, practically all of whom were interested in the use of medicinal plants for medicinal purposes (herbalists). Permission to conduct the study was obtained from local community leaders. A study guide identified the members of the target group as respondents who used ethnopharmacology for skin diseases and cosmetics. The study group formation employed the “snowball” technique. All safety measures were taken according to requirements of the pandemic situation (i.e., wearing face masks and gloves and maintaining a safe distance).

**Figure 2:**
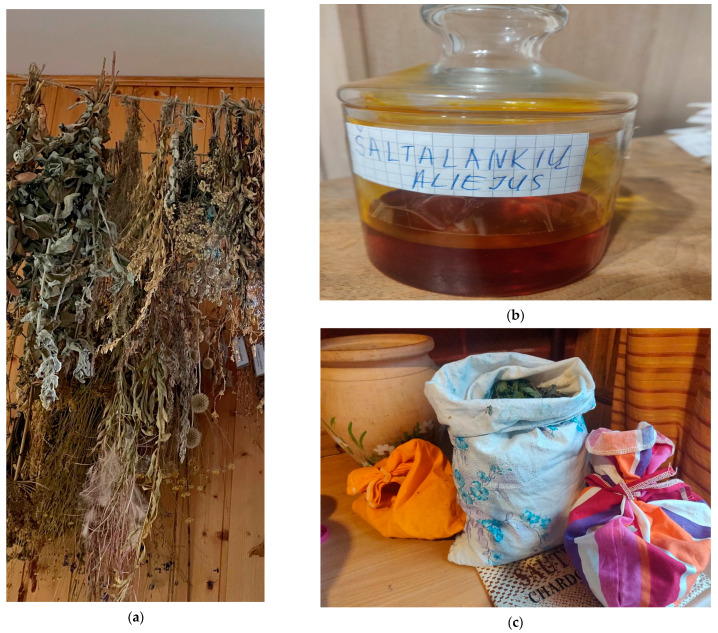
Preparation and storage conditions of medicinal raw materials used for skin diseases and/or cosmetics: (**a**) drying of various herbs; (**b**) prepared Sea buckthorn oil; (**c**) storage of dried plants in canvas bags.

The research method was a structured interview. It was carried out in two stages. During the first stage, using the prepared questionnaire File S1 (Supplementary Materials), the researcher wrote down the respondent’s answers. The prepared questionnaire consisted of 17 questions. The first stage started with the main questions for demographic data and closed questions to assess the source of ethnopharmaceutical information and to evaluate how many respondents chose to consult a healthcare professional (pharmacist or doctor) as a qualified consultant regarding ethnopharmacology for skin diseases and cosmetics. Much attention was paid to the sources of ethnobotanical knowledge obtained by the respondents. The aim of the second stage was to gather as much information as possible about products of natural origin, to identify medicinal raw materials used for medicinal and cosmetic purposes, their preparation methods, indications for use, doses, duration of use, and storage conditions (Figure 2). This information was obtained in the form of a free interview, and informants were allowed to speak spontaneously and without pressure. Interviews were voice recorded with permission from the respondents, and field notes were also taken and encoded. It was attempted to capture information about collected medicinal substances: where they were collected and how and under what conditions they were dried and stored. Respondents were visited a second time to supplement information if needed. The indications for skin diseases identified in the study were compared with the European Union herbal monographs by the Committee on Herbal Medicinal Products published by the European Medicines Agency (EMA) [20]. In this way, an attempt was made to determine the extent to which the indications for use in this study matched the indications approved in the EMA studies.

Taxonomic identification, botanical nomenclature and plant family assignment were performed based on validated databases, namely World Flora Online [21] and the Angiosperm Phylogeny Group IV [22]. Plant species were identified using writings on traditional Lithuanian flora [23,24,25].

The research data is stored in the Lithuanian Museum of the History of Medicine and Pharmacy of the Lithuanian University of Health Sciences.

## 3. Results and Discussion

### 3.1. Characteristics of Informants and Sources of Ethnopharmaceutical Knowledge

50 respondents were interviewed: 9 men (18%) and 41 (82%) women. The age of the respondents ranged from 23 to 94 years. 30% of the respondents were over the age of 65. When interviewing the respondents, it was important to determine whether the respondents were permanent residents of Šiauliai district and to determine if they were born there. Four respondents have changed their place of residence (moved to another district), but all of them named Šiauliai district as their place of residence. Therefore, we can assume that part of the ethnopharmaceutical knowledge collected in the study area is of local use. Respondents from 18 different residential areas of Šiauliai district were interviewed in this study. Most respondents lived in villages-Kužiai 15%, Voveriškiai 13%, Gruzdžiai 11% (Figure 1). 

The respondents’ education was also an important indicator in the survey. The oldest respondents in the survey, who were over 80 years old, had primary education 7 respondens (14%), secondary education 4 respondents (8%), vocational education 8 respondents (16%), higher education 7 (14%), and university education 24 (48%) respondents. This shows that in the first decades of the 21st century, 22% the of respondents did not have a profession and were primarily agricultural workers. A total of 20 (40%) respondents with vocational, higher and university education were engaged in agricultural activities. The other 19 (38%) respondents were not involved with agricultural work (studying or working in the city). 

Knowledge about the use of ethnopharmaceuticals for the treatment of skin diseases and for cosmetic purposes was obtained mainly from parents, grandparents, and relatives 45 (90%) of all respondents. Other sources of information were neighbors 27 (54%), the internet and television 26 (52%), doctors 9 (18%), pharmacists 25 (50%), and books 13 (26%). Every second respondent consulted a pharmacist on the use of ethnopharmaceuticals, which is a high percentage, considering that in our previous studies in Lithuania this percentage was incredibly low—0% [3], 8% [26] and 28% [27] of all respondents. A study on the attitude of the Lithuanian population towards phytotherapy revealed that only 1% of patients in all age groups turned to a pharmacist as a source of information on herbal medicine [28]. The reasons for not referring to a pharmacist were mainly distrust of pharmacists and their lack of knowledge about ethnopharmacology, and sometimes even the pharmacist’s ridicule of the patient for using such measures. In this study, due to the increase in the number of patients turning to a pharmacist for information on ethnopharmacology, the COVID-19 pandemic can be identified as the cause, due to it causing difficulties in reaching a physician or cosmetologist, leaving pharmacists as the most widely available healthcare professionals. On the other hand, the question arises as to whether the consultation and the knowledge of the pharmacist were sufficient and met the expectations of patients. 

### 3.2. Skin Diseases and Ethnopharmacology

**Figure 3:**
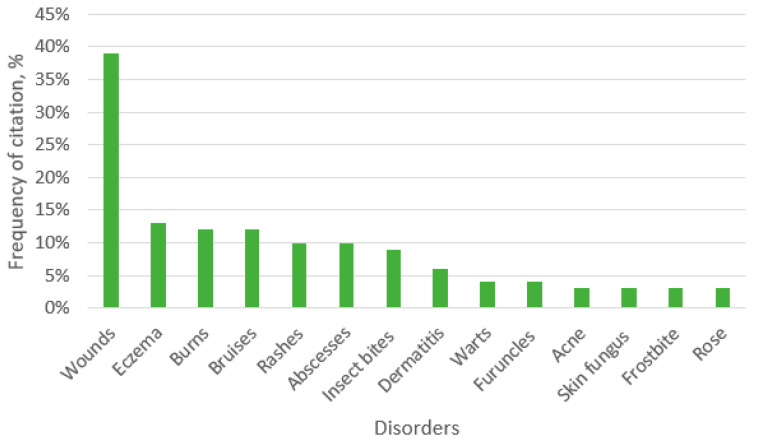
Ethnopharmaceutical preparations for skin disorders.

We documented 67 species of medicinal plants from 37 families used for skin diseases and cosmetics. All of the collected data are summarized in Table S1 (Supplementary Materials). According to our results, the most common indication of skin diseases were wounds (39%) (Figure 3).

**Figure 4:**
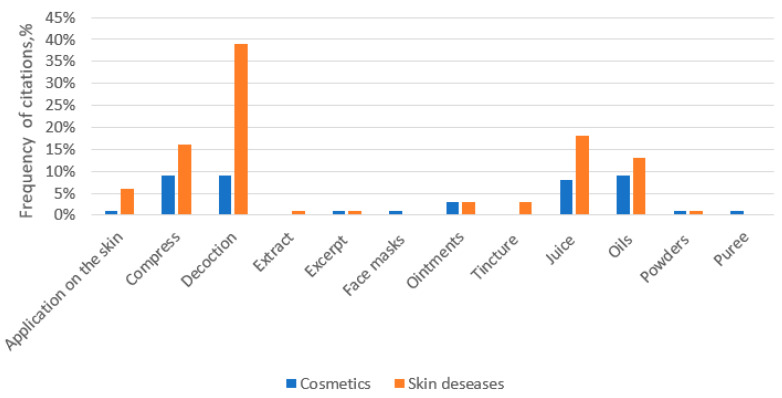
Methods of ethnopharmaceutical preparation for the treatment of skin diseases and cosmetics.

According to studies by other researchers, this is the most common indication of all skin diseases for which ethnobotanical measures are used [7]. For thousands of years, medicinal plants have represented the only remedy for wound care, and they still maintain an important therapeutic role. Of course, the main properties of plants used for wound care are antimicrobial and anti-inflammatory activities [29]. Although homemade herbal products in general are less expensive than modern treatments, they can lead to unexpected allergic reactions and side effects [30]. This is especially true when applied to the affected area of skin (excluding wounds, the main indications were eczema, burns, abscesses). In this study, all ethnopharmaceuticals were used externally, mostly as decoction, compress, juice, and oil applications (Figure 4). 

**Figure 5:**
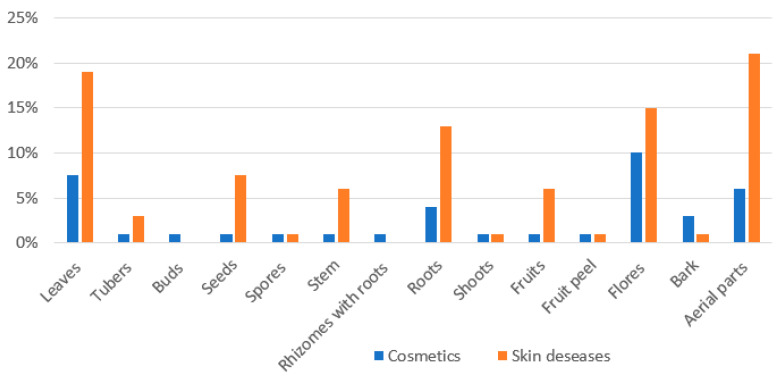
Parts of the plants used for ethnopharmaceutical preparations for skin diseases and cosmetics.

Although the most common method of administration is decoction usually used internally, in this case a decoction is prepared from plant-based raw materials and placed externally on the area affected by skin diseases. Only two types of plant raw materials were used internally, namely the decoction of *Viola tricolor* L. aerial parts for the treatment of furuncles, eczema, and rashes, the other internally used substance being a decoction made with roots of *Elytrigia repens* L., used to treat skin lesions, furuncles, and rashes. Decoction was the most common method of preparation in this study, using a soft above-ground part of the plant, so it is not surprising that the most used parts of the plant were aerial parts, leaves and flowers (Figure 5). The hard parts of plants (i.e., the seeds, stem, bark, and the fruits) were used less often, and less common preparation methods were used to prepare them, such as extraction with oil and tinctures. 

Jarič S and colleagues in their study on traditional wound-healing plants used in the Balkan region (Southeast Europe) [31] mentioned that the applications used for wound healing were external, in the form of infusions, decoctions, tinctures, syrups, oils, ointments, and balms, and were applied directly onto the skin, with *Plantago major* L. being the most popular medicinal plant.

In our study, the most cited plants for the care of skin diseases were *Plantago major* L. (80%) and *Chelidonium majus* L. (70%). *Plantago major* L. leaves have been used to treat wounds and traumas for centuries in almost all parts of the Europe [7,32]. According to our study, it was usually applied directly onto the skin. *Chelidonium majus* L. (70%) is a well-known plant in Lithuania, frequently the fresh juice of this plant is used to destroy warts. The plant accumulates alkaloids, so ingestion without medical supervision can be dangerous. In our study, the plant was used only externally to treat warts or psoriasis. *Chelidonium majus* L. has traditionally been used for the treatment of various inflammatory diseases and recently there were studies conducted on its use for atopic dermatitis [33] and antibacterial wound dressing applications [34]. 

In our study not only the frequency of use was identified, but also a comparative safety analysis with EMA monographs was carried out (Table S1). Modern pharmacological investigations have shown that many medicinal plants used in ethnobotanical traditions worldwide are being currently used in a very rational way, mainly synthesizing new and old information. Most clinical uses of medicinal plants have been similar to their traditional uses. However, there are some plants which have clinical uses that differ from traditional uses [7,35]. Despite their clinical effectiveness, a guarantee of the safety and quality of medicinal plants in developed countries is challenging as people increasingly return to herbal remedies refusing chemical drugs [27]. According to our study, respondents in Šiauliai district mentioned 67 species of medicinal plants from 37 different families used for the treatment of skin diseases and/or production of cosmetics. Only 24 species are described in the official herbal monographs of the European Medicines Agency (EMA) [20]. Sile I and colleagues from Latvia [36] have analyzed archives and found out that one of the most common health conditions were skin disorders. Analysis of EMA monographs showed that only 59 out of 211 taxa mentioned in this study are included in the official herbal monographs. EMA herbal monographs provide scientific information on safety and efficacy and deserve further exploration as traditional herbal medicines. In our study, 43 of 67 plant species were not included in the EMA monographs and only 14 species (21%) of all included species were used with EMA approved medical indications for the treatment of skin diseases. Other medicinal plants were used without EMA approved medical indications and were based solely on folk knowledge and experience in medicine. 

In the treatment of skin diseases, phytotherapy plays an important role, and other materials are also used as main or additional remedy bases. In our previous study regarding historical uses of the bee products, according to archival sources, honey and propolis were used to treat wounds and abscesses [37]. In this study, honey was used as an excipient (for example by preparing a home-made ointment, which is made by mixing pine shoots and honey) to treat psoriasis. For acne-prone skin, a facial mask is made by mixing a tablespoon of honey with 10 mL of almond oil and one egg yolk. To treat burns, 10 g of birch tar is mixed with 0.5 kg of honey and then the mixture is spread on the skin area affected by burns. Lard is often used as a base for the ointment. Melted hare fat is a popular remedy for treating splinters, and it is used as a poultice on the opposite side of where the splinter is. Cases where the whole animal is used instead of its products are usually only isolated occurrences (for example, the treatment of cold sores by rubbing a small frog’s abdomen on the affected area).

### 3.3. Ethnopharmacology and Cosmetics

**Figure 6:**
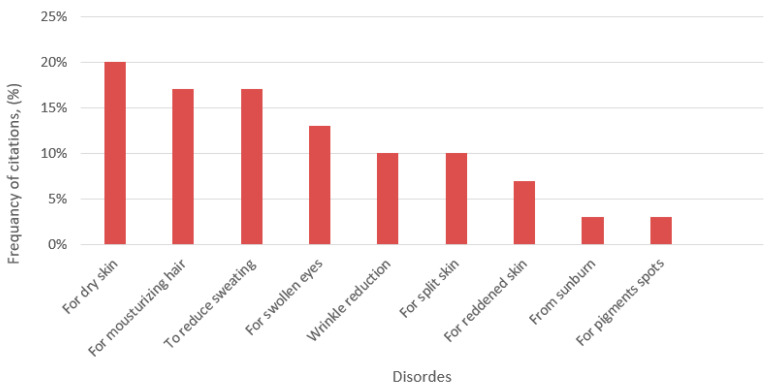
Ethnopharmaceutical usage for cosmetics.

Data on ethnopharmaceutical applications for cosmetic use made up 13.44% of our research (Table S1). Ethnobotanical studies combined with modern analyses of plants have a potential to enrich modern cosmetic products. Plants with cosmetic uses have often been neglected in ethnobotanical surveys which focus mainly on plants with medicinal and culinary uses [38,39,40]. Often, the target of researchers is the measures for treatment of skin conditions, but during the course of the study, it has been revealed that many of the plant preparations for therapeutic purposes were also used for cosmetic purposes, and making a clear distinction among the recorded preparations between cosmetics, cosmeceuticals and pharmaceuticals for the treatment of skin diseases is very problematic [41,42,43]. Although it may seem that the treatment of skin diseases is more important than skin care for cleanliness, prevention and restoration, beautification has also played an important role in rural communities. Studies show that the need for beauty treatments, including natural ones, has not diminished during the COVID-19 pandemic [44]. In our study during the COVID-19 pandemic in Lithuania, the main cosmetic uses were skin (20% of reports) and hair hydration (17%) and sweat reduction (17%) (Figure 6).

According to our knowledge, there are only some ethnobotanical studies in the world that research the medicinal properties of plants and their use for cosmetic purposes. Xenia J and colleagues [39] have conducted an ethnobotanical survey of cosmetic plants used in the Marquesas Islands (French Polynesia). The most referred application areas were the skin, the hair, and the genitalia, whereas the main cosmetic uses were perfume, hydration, medicinal care, and healing. In our study the most common areas of application were the face (40% of reports), the body (38%), the feet (16%), and the hair (7%). 15.5% of facial treatments were used to reduce eye swelling. 

If in Marquesas Islands the perfumed coconut oil, also known as monoi, was the main Marquesan cosmetic preparation used on skin and hair, in our study the main cosmetic preparation of *Aloe vera* L. (70%) juice was also used on skin and hair (Table S1). Our previous study on plants cultivated in Lithuania and the archival sources have revealed that *Aloe vera* L., usually called “the plant of elders”, was very popular to grow at home and was “always at hand when needed” [26,45]. Due to its special composition of amino acids, lectin, lipids, minerals, lactates, phenols, etc. and its soothing and cooling properties, aloe was not only used to improve the condition of the skin but also to treat it. It reduces itching and swelling, treats light cuts, bruises, eczema, has antibacterial, antifungal properties, and improves blood flow in the affected areas [46]. In our study, several cosmetic recipes are presented. For example, for acne, one tablespoon of crushed aloe mixed with three tablespoons of calendula oil. The mixture is applied on the face and left there for 20 min; an aloe mask used for application under the eyes, is made with 1 tablespoon of aloe juice, 1 tablespoon almond oil and half a grated cucumber, everything is mixed and applied under the eyes. The mixture is left there for 15–20 min. Burns are treated by applying the flesh of a peeled aloe leaf to the affected area. This plant is popular in today’s cosmetic products [47] and the modern consumer is usually aware of its beneficial properties for skin and hair. 

As in treatment of skin diseases, honey, pig fat, eggs, and buttermilk represented the most reported ingredients of animal origin in cosmetic applications. The face was washed with whey to remove freckles so that the skin was “clear”. The acid of ants was also used to remove freckles. This acid was acquired by throwing a scarf on an anthill, and when it dampened, the freckles were rubbed with it. Decoction, juice, and tincture with oil were most popular ways of preparation (Figure 4). As pointed out by Svanberg I [10], birch sap was the second most valuable product after wood to be found in the forest. It is renowned for antioxidative nutrients and high content of minerals. In northern Europe, birch sap has long been used not only as a food source but also for healing and cosmetics. The cosmetic use of birch sap was widespread in Estonia, where in the 19th century it was believed that washing the face with the first drops of birch sap will get rid of freckles and that the face will stay clear all summer. It was also used to treat skin diseases. In Lithuania, it was popular to rinse hair with birch sap. In this study, birch sap was also used to remove freckles and treat acne, blackheads, dry skin etc. Among the respondents, it was mentioned that the juice is frozen to ice and used to treat swollen eyes and to smoothen small wrinkles. To treat an oily scalp, two tablespoons of honey are mixed with four tablespoons of juice and a pinch of heated salt. The mixture is then diluted in half a glass of vodka, and it is then kept in a dark place for 10 days. The mixture is then rubbed into the oily scalp before washing. 

**Figure 7:**
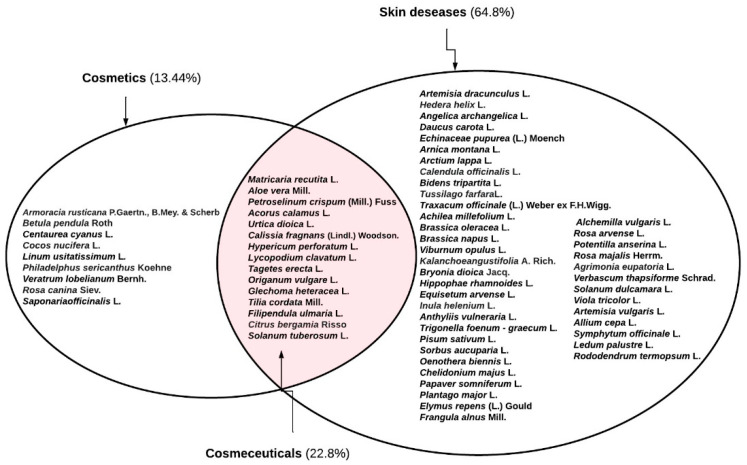
Use categories of medicinal plants.

The data from our study shows that the same plants are often used for cosmetic purposes and for the treatment of skin diseases. This distinguishes a separate group of cosmeceuticals. The term “cosmeceuticals” was first used by Raymond Reed in 1961 and the word and concept were further popularized by Dr Albert Kligman in the late 1970s [12]. Cosmeceuticals are both cosmetic and pharmaceutical preparations, intended to enhance health and beauty through ingredients that influence the skin’s biological function. These are cosmetic products that are not just used for beautification but also for different skin ailments, from acne-control and anti-wrinkle effects to sun protection. Medicinal plants as ingredients act not only as cosmetics, but also as pharmaceuticals [12]. In this study we highlighted medicinal plants used for cosmetics and for the treatment of skin diseases (Table S1). According to the results of our study, medicinal plants used for the treatment of medical disorders and for cosmetic purposes can be singled out as potential cosmeceuticals (Figure 7). 

## 4. Conclusions

The documentation of ethnopharmacological knowledge is important for the preservation of country’s cultural, social, and economic identity. Also, the application of modern methods of analysis enables the development of pharmaceutical and cosmetic products acceptable to the modern consumer. In terms of public health, not only ethnopharmaceutical knowledge and its application are relevant, but also the safety of “self-treatment” and recovery rituals for the treatment of skin diseases (which is especially relevant during the COVID-19 pandemic). Our study showed that only 21% of the indications for the use of ethnopharmaceuticals for the treatment of skin diseases coincide with the EMA assessment. The use of natural cosmetics and cosmeceuticals during the COVID-19 pandemic was popular in Lithuania. Improper use of ethnopharmaceuticals for cosmetic purposes and in the treatment of skin diseases can cause not only aesthetic but also health problems, and therefore ethnopharmacological knowledge must be critically assessed and based on scientific research. The ethnopharmacological knowledge of healthcare professionals (especially pharmacists) must be sufficient for the patient consultation and an adequate care.

## Data Availability

The data generated for this study are available from the authors upon request.

## Supplementary Materials

The following supporting information can be downloaded at: https://www.mdpi.com/article/10.3390/ijerph19074054/s1, File S1: Questionnaire, Table S1: Ethnopharmaceuticals for skin diseases and cosmetics in Siauliai district, Lithuania.
