# Supplementary material for: Ethnopharmacology for Skin Diseases and Cosmetics during the COVID-19 Pandemic in Lithuania
Source: Int J Environ Res Public Health. 2022 Mar 29;19(7):4054. doi: 10.3390/ijerph19074054 (PMC8998206; doi:10.3390/ijerph19074054)
Supplement: Supplementary file 1 [file ijerph-19-04054-s001.zip › File S1.pdf]

File S1. Questionnaire

1. Respondent's gender:
   - Female
   - Male
2. Respondent's age:
3. Education, profession :
   - Elementary
   - Basic
   - Secondary
   - Higher
   - Other
4. Residence:
5. How did you learn to treat with herbs?
   - From parents, grandparents
   - From neighbors, acquaintances
   - From books, newspapers
   - From radio, television, internet
   - From a family doctor, pharmacist
   - Other
6. Do you seek advice from your a pharmacist/doctor about using herbs to treat skin conditions?
   - Pharmacist
   - The doctor
   - I do not apply (why)
7. Are natural conditions and the time of collection important to you for collecting herbs?
   - Yes
   - No

8. What conditions of nature and time are important for you when collecting herbs?
   - Seasonality
   - Time of the day
   - Arrangement of celestial bodies, phases of the moon
   - Weather conditions (sunny, rainy or foggy days)
   - Other
9. Under what conditions and where do you store dried herbs?
10. What do you do with herbs that are no longer suitable for use?
   - Burned
   - Still consumed
   - Bury in the ground
   - You throw away
11. Do people appeals to you for help finding the herbal raw material they need?
   - Yes
   - No
12. Do you pass your experience of herbal treatment to other people?
   - Yes
   - No
13. What herbs do you collect?
14. What herbs do you grow yourself?

Structured part of the interview

15. For which skin deseases and which plants, their parts, preparation methods do you use?
16. For what cosmetic puposes and which plants, their parts, preparation methods do you use?
17. Compound recipes
