# Supplementary material for: Ethnopharmacology for Skin Diseases and Cosmetics during the COVID-19 Pandemic in Lithuania
Source: Int J Environ Res Public Health. 2022 Mar 29;19(7):4054. doi: 10.3390/ijerph19074054 (PMC8998206; doi:10.3390/ijerph19074054)
Supplement: Supplementary file 1 [file ijerph-19-04054-s001.zip › Table S1.pdf]

Table S1. Ethnopharmaceuticals for skin diseases and cosmetics in Siauliai district, Lithuania

Family	Botanical name	Local plant name	Used for skin diseases - L Used in cosmetics - K	Frequency of citations	Part used	Method of preparation	Therapeutic and cosmetic uses	EMA evaluation
Arecaceae	*Cocos nucifera* L.	Kokosas	K	10	Fruits	Oil	For dry hair<br>For dry skin	
Acoraceae	*Acorus calamus* L.	Ajaras	KL	15	Roots<br>Leaves	Decoction<br>Powder	From hair loss<br>For dry hair<br>Wounds healing	
Amaryllidaceae	*Allium cepa* L.	Cibulis, Cibule	L	25		Application on the skin	From insect bites<br>For abscesses	
Apiaceae	*Daucus carota* L.	Muorkva	L	10	Roots	Compress	For burns<br>Wounds healing	
Apiaceae	*Petroselinum crispum* (Mill.) Fuss	Petruška	KL	9	Leaves<br>Roots	Juice<br>Decoction	From bee and mosquito bites<br>For sunburns	
Apiaceae	*Angelica archangelica* L.	Švengaršvė	L	1	Roots	Decoction	For the treatment of acne and eczema	

Araliaceae	*Hedera helix* L.	Lipiki	L	2	Leaves	Decoction	For burns	EMA/HMPC/228356/2012 *Hederae helicis folium;* Cough and cold
Asphodelaceae	*Aloe vera* Mill.	Alijošius Elijuošius	KL	35	Leaves	Juice	Wound healing For frosbite shunvotes For dry skin	EMA/807482/2016 *Aloe vera folium;* For short-term use in cases of occasional constipation
Asteraceae	*Matricaria recutita* L.	Ramuliukai Ramunėlis	KL	15	Flowers	Compress	Wounds healing From swollen eyes	EMA/490629/2015 *Matricariae flos;* Gastrointestin al disorders; Cough and cold; Mouth and throat disorders; Skin disorders & minor wounds
Asteraceae	*Artemisia dracunculus* L.	Kėtis	L	1	Aerial parts	Decoction	From snake bites	
Asteraceae	*Bidens tripartita* L.	Šuniulai	L	3	Aerial parts	Decoction Compress	Wounds healing From rashes	
Asteraceae	*Arctium lappa* L.	Kibišas	L	17	Roots	Decoction	Seborians From insect bites From the incisions, From the burns From the bruises	EMA/HMPC/509409/2019 *Arctii radix;* Urinary tract and genital disorders; Loss of appetite; Skin disorders and minor wounds
Asteraceae	*Arnica montana* L.	Arnikas	L	21	Flowers	Tincture	Wounds healing From the bruises	EMA/654173/2016

								Arnicae flos; Skin disorders and minor wounds
Asteraceae	*Echinaceae pupurea* (L.) Moench	Ežiuoli	L	6	Aerial parts	Juice	Wounds healing	EMA/481797/2015 *Echinaceae purpureae herba;* Skin disorders and minor wounds; *Echinaceae purpureae radix;* Cough and cold; Skin disorders and minor wounds
Asteraceae	*Taraxacum officinale* (L.) Weber ex F.H.Wigg.	Pienė	L	4	Leaves	Application on the skin	From a bee bite	EMA/HMPC/579636/ 2008 *Taraxaci folium;* Urinary tract and genital disorders; Taraxaci radix cum herba;Gastrointestinal disorders;Loss of appetite;Urinary tract and gynecology disorders
Asteraceae	*Tagetes erecta* L.	Sekrečiukai	KL	2	Flowers	Excerpt	Wounds healing From skin fungus To reduce sweating	
Asteraceae	*Artemisia vulgaris* L.	Metėle	L	2	Stem	Juice	Wounds healing	

Asteraceae	*Inula helenium* L.	Debesyla	L	3	Roots	Decoction	For the treatment eczema and herpes<br>To reduce itching	
Asteraceae	*Centaurea cyanus* L.	Vasilka	K	3	Flowers	Compress	For balckened , bruised eyes<br>For inflammation of the eyelids<br>To reduce wrinkles	EMA/HMPC/228356/2012<br>*Centaurii herba;*Gastrointestinal disorders;Loss of appetite
Asteraceae	*Tussilago farfara L.*	Žalpusnis	L	7	Leaves<br>Flowers	Decoction	To treat shunvotes and abscesses<br>To treat rose<br>For beaten places<br>For the treatment of ulcerated skin	
Asteraceae	*– Calendula officinalis* L.	Naudatka	L	15	Flowers	Decoction<br>Compress	To treat eczema and dermatitis<br>To reduce rashes	EMA/HMPC/228356/2012<br>*Calendulae officinalis flos;*<br>Skin disorders and minor wounds;Mouth and throat disorders
Asteraceae	*Achilea millefolium L.*	Kruvinžolė	L	1	Flowers	Compress	For bruises, wounds, chest tears	EMA/HMPC/228356/2012

								*Millefolii flos;*Loss of appetite;Gastrointestinal disorders;Skin disorders and minor wounds;Urinary tract and genital disorders<br>*Millefolii herba;*Loss of appetite;Gastrointestinal disorders;Skin disorders and minor wounds;Urinary tract and genital disorders
Betulaceae	*Betula pendula* Roth	Svyruoklis	K	4	Bark<br>Buds<br>Leaves	Juice<br>Infusion	From freckles<br>To reduce acne and blackheads<br>For dry skin	EMA/HMPC/228356/2012<br>*Betulae folium;*Urinary tract and genital disorders
Boraginaceae	*Symphytum officinale* L.	Taukius	L	5	Roots<br>Leaves	Compress<br>Decoction	Wounds healing<br>For beaten places	EMA/HMPC/333915/2015<br>*Symphyti radix;*Pain and inflammation
Brasicaceae	*Brassica oleracea* L.	Kopūsts	L	20	Leaves	Sultys	To reduce redness of the face<br>To extract heat	
Brasicaceae	*Brassica napus* L.	Rapsas	L	3	Leaves	Decoction	To treat eczema and acne	
Brassicaceae	*Armoracia rusticana* P.Gaertn., B.Mey. & Scherb	Kriens	K	5	Roots	Puree<br>Compress<br>Kaukė	For pigmented spots<br>From pimples	

							From wrinkles	
Caprifoliaceae	*Viburnum opulus* L.	Putins	L	2	Fruits	Juice<br>Tincture	From rashes<br>For purulent skin	
Caryophyl-laceae	*Saponaria offici-nalis* L.	Muilažolė<br>Muilini	K	2	Flowers	Juice	To remove dirt<br>For dry skin	
Comme-linaceae	*Calissia fragnans* (Lindl.) Woodson.	Auksinis ūsas	KL	1	Leaves<br>Shoot<br>Stem	Juice<br>Ointment<br>Oil	For skin dermati-tis<br>From burns<br>From frostbite<br>From the bruises<br>From scars	
Crassulaceae	*Kalanchoe an-gustifolia* A. Rich.	Paleistuvė	L	3	Leaves	Juice	Wounds healing	
Cucurbitaceae –	*Bryonia dioica* Jacq.	Brienė	L	1	Roots	Compress	From the shocks	
Eleagnaceae	*Hippophae rham-noides* L.	Šaltakšnis	L	16	Fruits	Oil	From burns	
Equisetophyta	*Equisetum arv-ense* L.	Driežgeguž ė<br>Dirvinė eg-likė	L	2	Aerial parts	Extract<br>Decoction	From abraded blisters<br>Wounds healing	EMA/HMPC/278091/ 2015<br>*Equiseti herba;*Urinary tract and genital disor-ders;For supportive treatment of superficial wounds.
Ericaceae	*Ledum palustre* L.	Velnio šluota	L	5	Aerial parts	Decoction	From insect bites<br>From swelling<br>Wounds healing	

Ericaceae	*Rododendrum termopsum* L.	Rododendras	L	1	Aerial parts	Decoction	For the treatment of warts	
Fabaceae	*Trigonella foenum -graecum* L.	Uožragi	L	2	Seeds	Decoction	Wounds healing	EMA/HMPC/146221/ 2010<br><br>*Foenugraeci semen;*Skin disorders and minor wounds;Loss of appetite
Fabaceae	*Anthyliis vulneraria* L.	Perluotis	L	1	Flowers	Juice	Wounds healing	
Fabaceae	*Pisum sativum* L.	Žirneliai	L	5	Seeds	Compress	For abscesses<br>To treat shunvotes	
Urticaceae	*Urtica dioica* L.	Dilgelikė	KL	1	Aerial parts	Decoction	For dry skin<br>Wounds healing<br>For dry hair	EMA/HMPC/228356/ 2012<br>*Urticae herba*<br>Urinary tract and gental disorders<br>Pain and inflammation<br>Skin disorders and minor wounds
Hydrangeaceae	*Philadelphus sericanthus* Koehne	Jazminas	K	2	Flowers	Oil	For dry skin	
Hypericaceae	*Hypericum perforatum* L.	Joniukas<br>Marijos žolikė<br>Brandažolė	KL	6	Aerial parts	Oil	Wounds healing<br>For tears in the fingers	EMA/HMPC/101304/ 2008

								*Hyperici herba;*Mental stress and mood disorders;Skin disorders and minor wounds;Gastrointestinal disorders
Lamiaceae	*Glechoma heteracea* L.	Laukžolė	KL	2	Aerial parts	Compress	From the absceses<br>For split skin<br>For scratched skin	
Lamiaceae	*Origanum vulgare* L.	Raudonėlės	KL	4	Aerial parts	Decoction	Wounds healing<br>To treat shunvotes<br>Oily face and scalp rinse	
Lycopodiaceae	*Lycopodium clavatum* L.	Šarkažolė	KL	5	Spores	Ointment	For dry skin<br>For rashes	
Linaceae	*Linum usitatissimum* L.	Linelis	K	2	Seeds	Oil	Dried elbows	EMA/492394/2015 Corr.<br>*Lini semen;*Gastrointestinal disorders
Malvaceae	*Tilia cordata* Mill.	Liepžiedžiai	KL	4	Bark	Application on the skin<br>Compress	From scratches<br>From burns<br>For swollen eyes<br>For the treatment of roses	
Melanthiaceae	*Veratrum lobelianum* Bernh.	Čemeryčia	K	10	Rhizomes with roots	Juice	From lice	

Onagraceae	*Oenothera biennis* L.	Nakviša	L	11	Seeds	Oil	To treat eczema, dermatitis<br>From rashes	EMA/422144/2018<br><br>*Oenotherae biennis oleum;*Skin disorders and minor wounds
Papaveraceae	*Chelidonium majus* L.	Kapažolė<br>Karpžolė	L	35	Stem<br>Aerial parts	Juice<br>Decoction	For the treatment of warts<br>For psoriasis	
Papaveraceae	*Papaver somniferum* L.	Agona<br>Agūna<br>Aguoneli	L	2	Seeds	Oil	Wounds healing	
Plantaginaceae	*Plantago major* L.	Trauklapis<br>Gyslapis	L	40	Leaves	Application on the skin	Wounds healing	
Poaceae	*Elymus repens*(L.) Gould	Varputė	L	2	Roots	Decoction	Wounds healing<br>To remove furuncles<br>From rashes	
Rhamnaceae	*Frangula alnus* Mill.	Skirpstė	L	1	Fruits	Oil	For burns	EMA/HMPC/726261/2016<br>*Frangulae cortex;*Constipation
Rosaceae	*Alchemilla vulgaris* L.	Raskila	L	3	Leaves	Juice	From insect bites<br>Wounds healing<br>From abrasions	
Rosaceae	*Sorbus aucuparia* L.	Šermukšlė	L	5	Fruits	Juice	For the treatment of warts	
Rosaceae	*Rosa arvense* L.	Šunrožė	L	10	Flowers	Oil	To treat eczema and dermatitis	EMA/HMPC/137299/2013<br><br>*Rosae flos;*Mouth and throat disorders;Skin

								disorders & minor wounds
Rosaceae	*Potentilla anserina* L.	Žąsiažolė Sidabriukai	L	3	Aerial parts	Decoction	For rinsing wounds	
Rosaceae	*Rosa majalis* Herrm.	Erškėtis	L	4	Seeds	Oil	For the treatment of skin cracks, abrasions, bedsores, non – healing ulcers	
Rosaceae	*Filipendula ulmaria* L.	Vingiuorykštė	KL	3	Flowers	Decoction Powder	For rinsing wounds<br>For the treatment of furuncules<br>To treat eczema<br>Powder is spreaded on the scattered area	EMA/HMPC/228356/2012<br>*Filipendulae ulmariae flos;*Cough and cold;Pain and inflammation;*Filipendulae ulmariae herba;*cough and cold;Pain and inflammation
Rosaceae	*Agrimonia eupatoria* L.	Dirvuoli	L	2	Aeral parts	Decoction	Wounds healing	
Rosaceae	*Rosa canina* Siev.	Erškėtis	K	6	Flowers	Decoction	Skin rejuvenation and softening	EMA/HMPC/137299/2013<br>*Rosae flos;*Mouth and throat disorders;Skin disorders & minor wounds
Rutaceae	*Citrus bergamia* Risso	Bergamuotė	KL	2	Fruit‘s Peel	Oil	To remove scars	

							For the treatment of skin fungus	
Scrophularia-ceae	*Verbascum thapsiforme* Schrad.	Devynvaisti	L	2	Flowers	Decoction	To treat eczema	EMA/HMPC/228356/2012<br>*Verbasci flos;*Cough and cold
Solanaceae	*Solanum tu-berosum* L.	Bulbė	KL	10	Lumps	Compress	For burns<br>From swollen eyes	
Solanaceae	*Solanum dulcamara* L.	Karklavijas	L	1	Stem	Decoction	For furuncules	
Violaceae	*Viola tricolor* L.	Našlele	L	7	Aerial parts	Decoction	For furuncules<br>To treat eczema<br>For rashes	EMA/HMPC/131734/2009<br>*Violae herba cum flore;*Skin disor-ders and minor wounds
